# Relationship Between Body Mass Index (BMI) and Severity of Caries Among Primary School Children: A Scoping Review

**DOI:** 10.7759/cureus.71404

**Published:** 2024-10-13

**Authors:** Erika Norfitriah, Zahirrah Begam Mohamed Rasheed, Farinawati Yazid, Nurrul Shaqinah Nasruddin

**Affiliations:** 1 Dentistry, Universiti Kebangsaan Malaysia, Kuala Lumpur, MYS

**Keywords:** bmi, caries, caries index, pediatric, primary school children

## Abstract

The relationship between body mass index (BMI) and the severity of dental caries in children varies significantly across different age groups, with some studies showing favorable associations. At the same time, some found no or inverse links. This review examines the existing literature on the relationship between BMI and the severity of dental caries among primary school children specifically between the ages of six and 12. This review follows the preferred reporting items for scoping reviews (Preferred Reporting Items for Systematic Reviews and Meta-Analyses extension for Scoping Review (PRISMA-ScR)) guidelines. A comprehensive literature search was performed utilizing PubMed, Scopus, and Web of Science (WoS) to identify relevant studies published in English from January 2015 to June 2024. Studies on primary school children aged six to 12 years old, BMI, and severity of caries were included. Five hundred and seventy-seven articles were screened with 26 meeting the inclusion criteria. The majority of the studies were cross-sectional in design. The study identified a varied association between BMI and dental caries. Further understanding of this relationship can guide the development of preventive strategies and interventions that address childhood disproportionate BMI and dental caries, ultimately enhancing oral health and overall well-being in this population.

## Introduction and background

Dental caries remains a prevalent issue among children aged six to 12 years worldwide, with varying prevalence rates reported in different countries. Studies have shown that the prevalence of dental caries in deciduous teeth ranges from 95.5% to 100%, while permanent teeth range from 47.3% to 73.3%, with statistically significant differences [1,2]. The global burden of dental caries in children is further exacerbated by other factors such as socio-economic status, dietary habits, and oral hygiene practices, emphasizing the need for comprehensive preventive strategies to address this chronic childhood disease [2].

All teeth are susceptible to dental caries, but this susceptibility varies significantly based on factors such as location, morphology, structural composition, type of dentition, and genetics [3,4]. Teeth with deep pits and fissures are particularly prone to caries due to the increased likelihood of food and bacteria becoming trapped in these areas [4]. This risk is further heightened in posterior teeth in both adults and children, although anterior teeth in young children also show a high incidence of caries [5,6]. Once food becomes trapped, oral bacteria metabolize it, leading to the formation of a biofilm on the tooth surface, which plays a key role in the development of dental caries [7]. Although the presence of biofilm is essential to generate caries lesions, this sole factor alone is not sufficient to develop the disease due to its multifactorial nature [8]. 

Diagnosis of a disease involves a process that determines its presence using an objective method or system. In dental caries, various instruments have been utilized in clinical studies to assess the occurrence, including visual inspection and probing based on the World Health Organization (WHO) criteria, as detailed in Yang et al. (2015). The examination involved the use of a dental mirror and exploring probe to diagnose caries based on color, shape, or caries-like changes in teeth, with the assessment of decayed, missing, and filled teeth in both deciduous and permanent dentition using the dmft and Decayed, Missing and Filled Teeth (DMFT) indices, respectively. The DMFT index is a commonly employed metric for evaluating dental caries, especially in epidemiological studies, and is used for assessing the condition of an individual's teeth [9]. It takes into account both current and prior instances of tooth decay [10]. Additionally, studies have also highlighted the importance of calibrated professional dentists for accurate assessments with good inter-examiner agreement indicated by high kappa values [1,11].

Since health problems are largely associated with growth and development, a number of research studies have investigated the relationship between weight status and caries using BMI [12]. BMI is a numerical measurement calculated from a person’s weight and height, serving as a valuable tool in population studies to classify body mass into categories related to health concerns [13]. Some studies showed an association between dental caries and BMI in children, in which findings showed a positive correlation between the DMFT caries severity index and BMI, especially in overweight children while some research suggests that there may not be a direct relationship [11,14-17]. Furthermore, anthropometric measurements have also been correlated with dental caries, highlighting the importance of understanding the interplay between nutritional status and oral health in children [18,19]. This scoping review aims to comprehensively identify, describe, and map the existing literature on the relationship between BMI and dental caries among primary school children.

## Review

Methods

Data Sources and Search Strategy

To comprehensively map the current studies in this field and discover any gaps in knowledge, a scoping review was conducted. The objective of the review was to address the research query "What is the relationship between BMI and the severity of dental caries among primary school children by systematically mapping the existing literature?" Thematic and quantitative analysis were used to translate the findings. This study examines primary school children (populations) and evaluates BMI (interventions). There was no comparison group (C), and the main outcome being measured was the severity of caries (outcomes). A thorough literature search was performed on electronic databases such as PubMed, Scopus, and Web of Science (WoS) to identify pertinent research published in English using specific keywords such as "Body Mass Index," "children caries," "children tooth decay," and "caries index." The search was restricted to publications published from January 2015 to June 2024, utilizing the "AND" and "OR" operators.

Employing study designs such as cross-sectional study, cross-sectional and descriptive, descriptive-analytical, case-control, analytical cross-sectional, secondary analysis of a cross-sectional, and observational follow-up are considered valid since they provide robust evidence of the relationship. The selection of studies was also restricted to free-access articles to enable comprehensive examination and analysis of the findings and methodologies. The research team utilized the Preferred Reporting Items for Systematic Reviews and Meta-Analyses extension for Scoping Review (PRISMA-ScR) standards to create our protocol, making necessary adjustments.

Eligibility and Selection Strategy

The search findings obtained from the three databases (PubMed, Scopus, and WoS) as tabulated in Table 1 were exported to the reference-management software EndNote version 20, and the duplicates were removed using the “Find duplicates” function to facilitate the process of selecting potential research for inclusion. The remaining articles were exported into Microsoft Excel (Microsoft Corporation, Redmond, Washington, USA) for the title and abstract screening performed by two independent reviewers (EN and ZBMR), guided by the eligibility criteria for this review. This stage was crucial in evaluating the pertinence of the studies to the research problem, guaranteeing that only papers containing substantial information concerning the BMI and severity of children's caries were kept. A thorough and complete examination of the remaining papers was done, including a study of the entire manuscript. This stage involved a comprehensive assessment of the whole subject matter of every research article to confirm its suitability for inclusion in the review. Articles that were not considered fully accessible were rejected at this step, as comprehensive content was necessary for a comprehensive analysis.

**Table 1:** Literature search strategy for the study selection process

Database	Search string
Pubmed	((BMI) OR (body mass index)) AND ((children tooth decay) OR (dental caries) OR (cavities) OR (early childhood caries) OR (pediatric dental caries) OR (tooth cavities) OR (caries lesions) OR (dental decay) OR (decay lesions)) AND (caries index) Year: 2015-2024 Text Availability: Free Full Text Article Language: English Species: Human Total: 88
Scopus	((BMI) OR (body mass index)) AND ((children tooth decay) OR (dental caries) OR (cavities) OR (early childhood caries) OR (pediatric dental caries) OR (tooth cavities) OR (caries lesions) OR (dental decay) OR (decay lesions)) AND (caries index) Year: 2015-2024 Subject Area: Medicine Document Type: Article Language: English Open Access: All Open Access Total: 206
Web of Science	((BMI) OR (body mass index)) AND ((children tooth decay) OR (dental caries) OR (cavities) OR (early childhood caries) OR (pediatric dental caries) OR (tooth cavities) OR (caries lesions) OR (dental decay) OR (decay lesions)) AND (caries index) Publication Years: 2015-2024 Document Types: Article Language: English Open Access: All Open Access Total: 99

Data Charting

The process of extracting data was systematically conducted for the studies that fulfilled the inclusion criteria, with a specific focus on key aspects that are pertinent to the study question. The eligibility requirements were determined according to the PECOS framework. (i) Population. Studies that encompassed schoolchildren aged six to 12 years old of any ethnic background or gender were included. Similarly, studies that examined youngsters without any underlying health conditions were also included. (ii) Exposure. The studies that were included were required to include data on BMI. (iii) Results. Included in the studies were investigations examining the correlation between being underweight and/or overweight and oral health. The study incorporated oral health indices such as decayed, missing, and filled (permanent) teeth (DMFT); decayed, missing, and filled (permanent) teeth surfaces (DMFS); and decayed, removed, and filled primary teeth (deft). (iv) Research methodology. This review evaluated many types of studies, including cross-sectional study, cross-sectional and descriptive, descriptive analytical, case-control, analytical cross-sectional, secondary analysis of a cross-sectional, and observational follow-up.

Result

**Figure 1:**
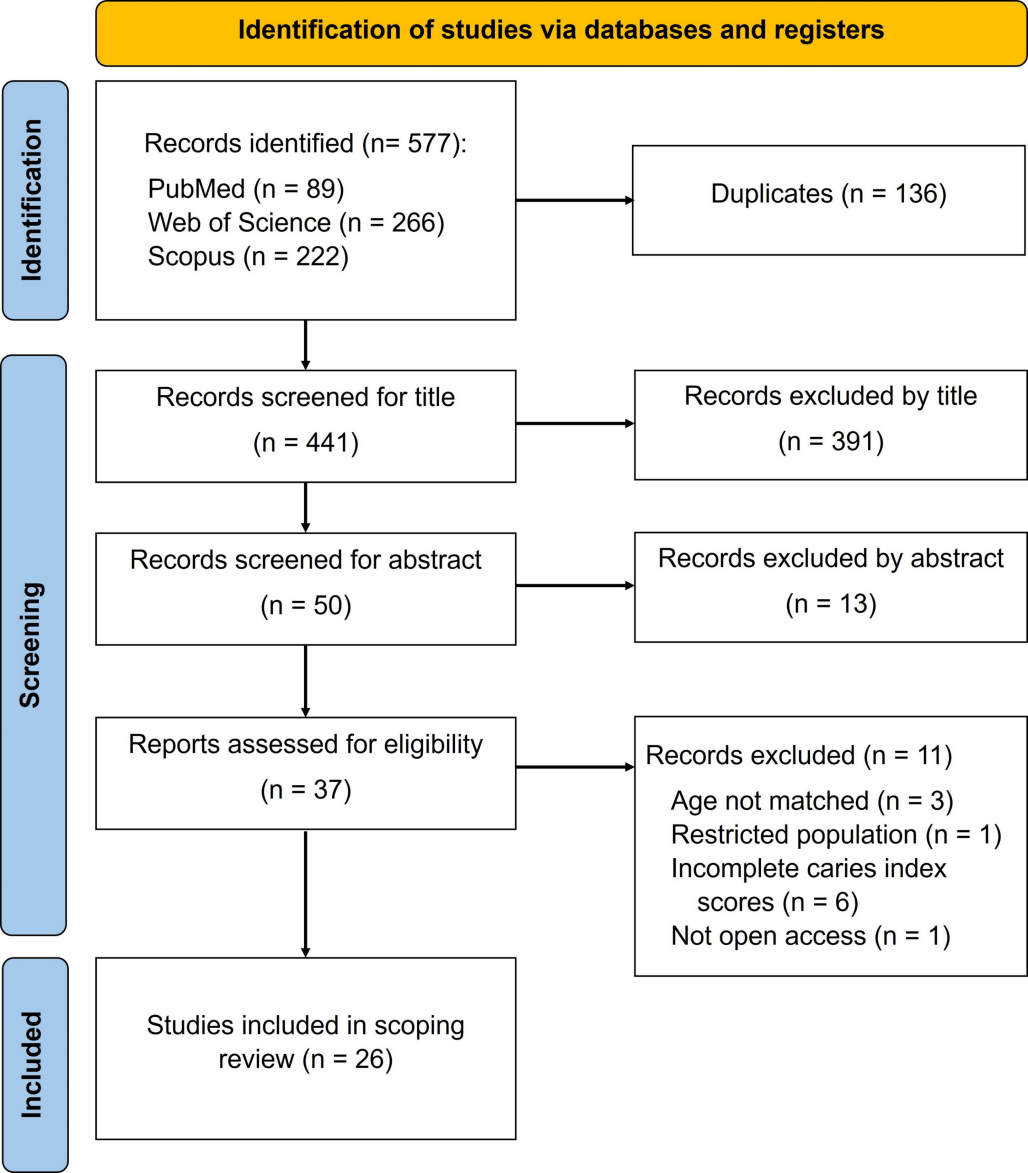
Flowchart of the study selection process

The bibliographic search using the specified search terms as in Table 1 yielded 577 potentially relevant articles. Initial screening excluded 136 articles due to duplication, and 404 articles were removed based on the titles and abstracts for reasons such as review papers, irrelevant parameters, or not being primary school children as one of the inclusion criteria. The remaining articles were subjected to a defined selection process, whereby 11 were further removed because these studies used an age range not specified in the inclusion criteria, a non-caries/incomplete caries index, no open access, and a restricted population, thus resulting in a final selection of 26 articles. The search flowchart is depicted in Figure 1.

The details of the author, publication year, study design, sample size, and outcome of each study are summarized in Table 2. A total of 15 studies performed cross-sectional, one was cross-sectional and descriptive, one was descriptive analytical, one was case-control, two were analytical cross-sectional, one was a secondary analysis of a cross-sectional, one was an observational follow-up, one was descriptive cross-sectional, and three studies have not mentioned the study design [1,2,11,14-36]. The lowest sample size was 56 individuals, and the highest was 32,461 individuals. Both male and female genders were counted to be included in the study analysis.

**Table 2:** Summary of included studies (n=26)

Author (publication year)	Population	Sample Size	Country	Study mode	Outcomes
Bulut, et al. (2020) [1]	6-11 years old	178	Turkey	Case-control	(i) Statistically significant association between BMI and caries in the permanent dentition. (ii) No significant association between BMI and caries in the primary dentition. (iii) No significant difference in caries prevalence with gender. (iv) No significant difference in caries prevalence with parent’s educational level.
Kapil, et al. (2023) [2]	6-12 years old	400 (132 females, 268 males)	Faridabad City, Haryana	Cross-sectional	(i) Statistically significant association between caries prevalence with BMI in the permanent teeth but not in deciduous teeth. (ii) Highest caries prevalence in the deciduous teeth in upper-lower and lower-middle-class children. (iii) Highest mean dmft score in deciduous teeth in underweight children and overweight children in the permanent teeth. (iv) High mean dmft and mean DMFT with tooth brushing. (v) Highest mean dmft score with frequent dental visits compared to no dental visits. (vi) Lowest mean DMFT score with frequent dental visits compared to one dental visit. (vii) Significant correlation of oral hygiene practices, beverages, drinks, chips, candy, and tea/coffee and BMI with caries prevalence. (viii) Higher rate for caries in females.
Yang, et al. (2015) [11]	8 years old	744 (384 males, 360 females)	China	Cross-sectional	(i) Statistically significant difference of dmft/(dmft+DMFT) score with BMI. (ii) Underweight children had the highest dmft/(dmft+DMFT) scores. (iii) BMI had an inverse relationship with dmft/(dmft+DMFT) scores. (iv) No significant difference between male and female and caries prevalence.
Alsweed, et al. (2023) [14]	9-12 years old	524	Saudi	Descriptive cross-sectional	(i) Statistically significant higher DMFT scores in the overweight children compared to normal and underweight children. (ii) Positive correlation of DMFT and BMI with caries severity.
Kotha, et al. (2022) [15]	6-12 years old	400 (243 females, 157 males)	Riyadh	Cross-sectional	(i) Higher prevalence of caries in primary dentition compared to permanent dentition. (ii) Significant relationship between BMI categories with both decayed and missing teeth, particularly in the underweight, overweight, and obese categories. (iii) Decayed teeth more prevalent in underweight and overweight. (iv) DMFT scores slightly more in underweight children. (v) Significant positive association of BMI with DMFT.
Reddy, et al. (2019) [16]	6-12 years old	1500 (819 females, 681 males)	India	Cross-sectional	(i) Lower-class children had more caries prevalence. (ii) Statistically significant caries occurrence in underweight compared to normal weight and obese groups. (iii) Less risk of caries in normal weight children compared to underweight children. (iv) Less risk of caries in overweight children compared to underweight children. (v) Statistically significant difference between sugar exposure and caries in primary and permanent teeth. (vi) Positive correlation of height, weight, BMI, weight for age, skinfold thickness subscapular and triceps, body fat percentage, waist circumference, hip circumference, and mid-upper arm circumference with caries in primary and permanent teeth. (vii) Negative correlation and significant difference in height for age and BMI for age with caries in primary and permanent teeth.
Aoun, et al. (2023) [17]	12 years old	782 (360 females, 422 males)	Libya	Secondary analysis of a cross-sectional study	(i) No significant association of anthropometric measures with caries. (ii) Higher BMI in females and self-reported regular sugary drinks.
Özel, et al. (2024) [18]	6-12 years old	210 (105 females, 105 males)	Turkey	-	(i) Significantly lower DMFS with white cheese daily consumption. (ii) Significantly higher DMFT and dmfs scores with biscuits daily consumption. (iii) Significantly higher DMFS scores with sweets and spreadable chocolate daily consumption. (iv) Correlation between height and body weight with DMFT-dmft and DMFS-dmfs scores. (v) Positive correlation of BMI with DMFT and DMFS but negatively correlated with dmft. (vi) Significant association of maternal education level, frequency of sugar intake, and body fat ratio with caries presence. (vii) Higher chance of caries with daily sugar consumption.
Dikshit, et al. (2018) [19]	7-12 years old	251 (118 females, 133 males)	Nepal	Cross-sectional	(i) No statistically significant difference between nutritional status and caries. (ii) Statistically significant difference between caries with gender and age. (iii) No statistically significant relationship between DMFT with BMI and age. (iv) Significantly higher mean DMFT in females than males.
Al-Janabi WH (2019) [20]	7-11 years old	56 (33 females, 23 males)	Iraq	Cross-sectional	(i) High significant correlation between BMI and dmfs score. (ii) No significant difference in dmfs scores between males and females.
Barbosa, et al. (2021) [21]	8-11 years old	353 (170 females, 183 males)	Brazil	Cross-sectional	(i) Association between caries with overweight and obese children. (ii) No significant difference in ICDAS for gender.
Fernández, et al. (2017) [22]	8-12 years old	1210 (636 females, 574 males)	Brazil	Cross-sectional	(i) Inverse relationship between obesity/overweight children and caries. (ii) Lower prevalence of caries in overweight/obese children. (iii) Less dental caries experience in overweight/obese children with 300 minutes per week of physical activity.
Goodarzi, et al. (2019) [23]	10-12 years old	416 females	Iran	Cross-sectional	A significant association between dental caries and high BMI.
Leila Basir, et al. (2021) [24]	6-12 years old	300 (138 females, 162 males)	Iran	Cross-sectional	(i) Significantly higher mean weight in high caries risk children. (ii) Significantly lower mean DMFT scores in low caries risk children. (iii) Significantly higher BMI in high caries risk children. (iv) Direct and significant relationship between DMFT and BMI with caries risk. (v) No significant difference in caries risk for parent’s education and gender.
Liang, et al. (2016) [25]	7-9 years old	32,461 (14,778 females, 17,683 males)	China	Cross-sectional	(i) Negative correlation between caries with BMI. (ii) Decreased odds for caries prevalence of primary teeth in overweight and obese children. (iii) Significantly higher mean dmft in males compared to females.
Nkambule, et al. (2019) [26]	12 years old	440 (207 females, 233 males)	South Africa	Cross-sectional	(i) No significant association between caries prevalence with BMI, diet, or socioeconomic. (ii) Significant association between no caries with eating less than three sweets a day.
Serrano-Piña, et al. (2020) [27]	8-12 years old	331 (171 males, 160 females)	Mexico	Cross-sectional	(i) Higher caries experience in underweight and normal weight children. (ii) Greater caries experience in the permanent teeth in overweight children. (iii) Greater caries experience in the primary teeth in underweight children. (iv) Negative correlation between BMI-for-age with caries.
Yen, et al. (2021) [28]	6-12 years old	569 (286 females, 283 males)	Taiwan	Cross-sectional	(i) Significant negative correlation between anthropometric measures with deft scores. (ii) Significant positive correlation between anthropometric measures with DMFT scores and age. (iii) Significantly lower deft score in obese children than normal/underweight children. (iv) No significant association between weight status with DMFT or deft scores. (v) Significant association between father’s educational level with caries in primary teeth. (vi) Significant association between gender and father’s and mother’s educational level with caries in permanent teeth.
Younus, et al. (2020) [29]	6 years old	218 (116 females, 102 males)	Iraq	Cross-sectional	(i) Highest DMFT index in underweight children. (ii) Lowest caries index in normal BMI children. (iii) No significant relationship between BMI and DMFT.
Amit, et al. (2024) [30]	6-11 years old	79 (47 females, 32 males)	-	Cross-sectional and descriptive	(i) Statistically significant positive correlation between BMI and caries. (ii) Statistically significant relationship between caries severity with average consumption of milk and dairy products and intake of fruits and vegetables.
Soleimani, et al. (2023) [31]	6-12 years old	300 (168 females, 132 males)	Tehran	Descriptive analytical	(i) No association between BMI with dmft and DMFT. (ii) Significant correlation between BMI and rate of caries in permanent teeth. (iii) Increased dmft index with higher consumption of sugary snacks. (iv) Decreased dmft with increased frequency of tooth brushing. (v) Significantly decreased DMFT with improved tooth brushing frequency and dental visits. (vi) Father’s educational level affects caries prevalence.
Anzar, et al. (2021) [32]	6-9 years old	376 (189 females, 187 males)	-	Analytical cross-sectional	(i) Significant association between caries with height-for-age Z-score, weight-for-age Z-score, and BMI-for-age Z-score. (ii) Inverse relationship between anthropometric measures with low, medium, and high caries categories.
Bhayat, et al. (2016) [33]	12 years old	402	Saudi	Analytical cross-sectional	(i) Significantly inverse relationship between DMFT and BMI. (ii) Significantly higher caries prevalence between normal BMI and DMFT score compared to overweight and obese children. (iii) Significantly lower BMI in males with severe caries than mild or no caries. (iv) 2-fold risk of developing caries in normal and underweight children compared to overweight and obese children. (v) No significant association between dietary habits with caries.
Sánchez-Pérez, et al. (2021) [34]	6 years old	201 (104 females, 96 males)	Mexico	Observational follow-up survey	(i) No significant association between caries prevalence and incidence with BMI. (ii) Highest caries index in children with malnutrition.
Vardhana, et al. (2023) [35]	6-12 years old	500	United States	-	(i) No significant difference between deft and DMFT scores with BMI for all age categories. (ii) No significant difference between the consumption of supplements on a daily basis with BMI for all age categories.
Vasconcelos, et al. (2019) [36]	10-12 years old	197 (101 females, 96 males)	Brazil	Studied population	Dental caries is associated with obesity.

Only one study has employed the International Caries Detection and Assessment System (ICDAS) as a parameter for caries diagnosis. Nine articles exclusively utilized the DMFT index as an indicator [1,2,15,16,18,20,21,24-26]. One study employed only the dmft index [23]. Fourteen articles used both the dmft/deft and DMFT indices as indicators [3-8,10-14,17,19,22]. One study utilized both the DMFT index and the PUFA (Pulpal involvement, Ulceration, Fistula, and Abscess) index as parameters [9].

Various outcomes were reported on the relationship between BMI and dental caries among primary school children. From the total of 26 studies, 10 studies showed a positive relationship between BMI and caries severity and six studies showed an inverse or negative relationship [11,14-16,19-25,27,30,32,33,36]. Nonetheless, another seven studies failed to demonstrate any relationship between these two parameters [1,2,17,18,26,28,29,31,34,35]. However interestingly, Özel, IÇ, et al., Bulut H, et al., and Kapil D, et al. studied the relationship between BMI and dental caries in both the primary dentition and the permanent dentition. Findings from these studies reported the same outcome on the positive correlation between BMI and dental caries on permanent teeth with a negative correlation in the primary teeth.

Discussion

In this review, although initially, 441 articles passed for review based on the title and the abstract, most of the articles focused on adults rather than children. This implies that caries research thus far has been focusing on teenagers and adults due to the high global burden of dental caries compared to children [37]. Nonetheless, around 530 million children worldwide are affected by oral disease, with dental caries being the most prevalent [38,39]. This statistic is highly significant and should not be overlooked, which highlights the need for rigorous research on children with caries. 

BMI and Caries Relationship

Dental caries is a multifactorial disease that can occur with the interplay of BMI as one of the main roles. This is the first study reviewing the connection between BMI and dental caries among primary school children with a conflicting relationship between the BMI categories of normal, underweight, and obese/overweight status and dental caries experience, prevalence, risk, or occurrence. According to Reddy, et al., Kotha, et al., Serrano-Piña, et al., Bhayat, et al., Younus, et al., and Yang, et al., underweight primary school children had the highest caries disease compared to other BMI categories [11,15,16,27,29,33]. On the other hand, Vasconcelos, et al., Alsweed, et al., Bulut, et al., Barbosa, et al., and Yen, et al. reported that obese/overweight children had the highest caries disease while Liang, et al. and Yen, et al. found lower odds and deft scores compared to normal and underweight children, respectively [1,14,21,25,28,36]. Conversely, Fernández, et al. study, which only had an obese population, reported a low caries prevalence [22]. The remaining studies only showed a general association without specifying the BMI categories that are directly associated with BMI.

Dietary Factors

Contradictory results found in this review could be due to several factors such as the geographical locations of the studies, the socioeconomic status, dietary habits, and active physical activity of the studied population. The high caries status in the underweight population in the Reddy, et al. study is because the sample size was collected in rural areas with a possibility of chronic malnutrition status [40]. This early stage of malnourishment is believed to cause enamel hypoplasia and salivary hypofunction, which leads to a decreased salivary flow rate and an increase in plaque accumulation, ultimately causing dental caries [41]. Surprisingly, studies conducted in Saudi Arabia, a high-income country, and China, an upper-middle-income country, have a similar outcome, for which all authors believed that the results could be due to the unique dietary patterns [11,15,33]. A poor diet or increased caries could have resulted in dental pain, impacted chewing and eating functions, and reduced nutritional intake, thus lowering their BMI [42]. However, dietary habits could not be confirmed as factors in these studies since this parameter was not accounted for. Aside from poor diet, untreated early childhood caries (ECC) children may have been included in the underweight population in these studies. The pain and discomfort from untreated dental decay tend to lower the appetite of these children thus they generally exhibit a significantly lower body weight [43].

Sedentary Lifestyle and Oral Health

In contrast to underweight, overweight or obesity is commonly known as the main predisposing factor for dental caries [44]. Nevertheless, Fernández, et al. in their study demonstrated low caries prevalence among the obesity population, which shows the difficulty and complexity of speculating the mechanism by which obesity and dental caries were not associated. Active physical activity performed by the population in the study explained the negative correlation, although both the development of obesity and dental caries are associated with high consumption of refined carbohydrates and sugar [45]. Therefore, it is plausible that the dietary pattern of the population avoids sugary intake but rather fatty foods since it is evident from Reddy, et al., Özel, et al., Kapil, et al., Soleimani, et al. and Nkambule, et al. studies that sugary food consumption is linked with dental caries. The geological location of the study, which takes place in Brazil, an upper-middle-income country, better explains the conflicting result. Despite the possibility that the population has an inappropriate nutritional status, these children have access to better education on oral health behaviors such as frequent dental visits and tooth brushing, thus leading to an overall lower incidence of caries [46]. Two studies by Soleimani, et al. and Kapil, et al. corroborated this notion with their findings that DMFT scores among their population decreased with frequent dental visits [2,31]. Even so, a contrary finding was gathered on the teeth brushing frequency from both studies, whereby DMFT scores decreased in Soleimani et al. but increased in Kapil et al. A possible explanation for the increased DMFT is the use of non-fluoridated toothpaste since fluoride has been shown effective in preventing dental caries in a meta-analysis study [47]. However, the type of toothpaste was not mentioned in the study.

Gender and Socioeconomic Factors

Gender and the prevalence of dental caries have long been associated with evidence showing that females have a higher caries experience compared to males across many populations [48-50]. Most of the studies in this review lack discussion of this parameter in their populations; however, several studies demonstrated a similar outcome within their populations, with one study showing a different outcome in males [2,19,25.27]. Typically, both males and females have a similar timeframe during childhood for permanent teeth eruption but the earlier eruption of canines and first premolars in females may expose the teeth longer to cariogenic products in the oral cavity [51]. Recent studies on metagenomics also revealed the functional role of individual genomes in dental caries variability in terms of gender and ethnicity thus emphasizing the involvement of genetics in the susceptibility of dental caries [3,52]. Furthermore, the relationship between parental education and caries risk in children is also important, aligning with the reported data in this review [53,54]. Parents who are educated may have better oral health awareness and engage in more preventive behaviors such as regular dental visits and proper oral hygiene practices with their children.

Caries Index Assessment

The age of six to 12 is the period during which deciduous teeth begin to fall out and permanent teeth start to emerge [55]. Following a recommendation by WHO, dmft/DMFT caries index is the most used to measure caries experience in permanent teeth (DMFT) and in primary (or deciduous) teeth (dmft). Notably, only three studies highlighted DMFT and dmft index scores in their findings in relation to BMI while most studies evaluated only one dentition [1,2,18]. Assessing both dentitions separately is preferred to distinguish the presence of healthy teeth in the caries evaluation instead of using DMFT/dmft mean values in mixed dentition since it is not highly accurate due to the wide range of age and the duration of the teeth in the mouth [56]. While the majority of the research utilizes DMFT criteria for diagnosis of caries, only one study used ICDAS as their diagnostic method [21]. ICDAS has been demonstrated to present a greater sensitivity and specificity compared to the dmft/DMFT index in detecting caries [57]. Further comparison between these two indexes showed that a lot of important information is lost because most of the lesions are non-cavitated while dmft/DMFT index only evaluates cavitated lesions with exposed dentin [58]. Most of the time, the only possible treatment is invasive ending either with an operative treatment or a surgical procedure for which the children are submitted to the dentist. In contrast, an early detected lesion, preferably using ICDAS, will allow non-invasive or minimally invasive actions to be performed by the dentist during a dental visit.

Limitations

This review has some limitations. The results presented in this review were entirely dependent on the three databases that are mostly adopted in medical science, while some grey literature may not be included. Therefore, it is possible that another study using different search terms and methods may arrive at different findings. The majority of the studies also conducted a cross-sectional design whereby a cause-and-effect relationship may not be fully established. It is also challenging to establish the relationship or the association since these articles lack in categorizing the children population into early mixed dentition, late mixed dentition, and permanent dentition phases. This unavailability may introduce bias in determining the final outcomes. The recommendation is longitudinal follow-up studies may be required to assess the impact of caries on primary school children with complete and comprehensive assessments of all factors including genetic parameters for a better understanding since dental caries is a multifactorial disease.

Future Direction

The current generation is marked by significant technological advancements, particularly in the realm of artificial intelligence (AI). The ongoing development of AI tools presents immense potential for improving children's oral health. These technologies can be leveraged to enhance parental guidance in preventing dental caries. AI models, such as ChatGPT, can process vast datasets related to dental caries and BMI, enabling the delivery of personalized advice tailored to individual needs [59]. For instance, ChatGPT could offer parents specific recommendations based on their child's dietary habits, activity levels, and existing health conditions in real time. Furthermore, AI tools can serve as educational platforms, providing parents with critical information on maintaining a healthy BMI and promoting good oral hygiene using evidence-based strategies. Beyond the individual level, these systems can be integrated into healthcare providers' databases to monitor children's health metrics over time, allowing for proactive management of dental and weight-related issues and ensuring timely interventions. At a community level, AI tools can assist in designing effective public health programs by analyzing demographic data to identify areas with higher rates of dental caries or obesity. By harnessing big data and offering personalized guidance, these technologies hold the potential to improve health outcomes and empower parents with the knowledge necessary to promote healthier lifestyles for their children [60].

## Conclusions

In conclusion, this scoping review reveals conflicting findings regarding the relationship between BMI and dental caries in primary school children. The association may be constrained by the lack of consideration of other significant factors that contribute to caries development. Further research is necessary to enhance the reliability of the findings and address current knowledge gaps, particularly by prioritizing longitudinal studies to monitor trends over time and adopting a multifactorial approach that takes into account dietary habits, socioeconomic factors, physical activity, genetic predisposition, and access to dental care. Standardized methods for assessing BMI and detecting dental caries are crucial for ensuring consistency in future studies. Additionally, interventional research focusing on nutrition, physical activity, and oral health may offer effective preventive strategies, while integrating AI and big data analysis could improve risk prediction and facilitate early intervention.
